# Decreased resting-state alpha-band activation and functional connectivity after sleep deprivation

**DOI:** 10.1038/s41598-020-79816-8

**Published:** 2021-01-12

**Authors:** Jintao Wu, Qianxiang Zhou, Jiaxuan Li, Yang Chen, Shuyu Shao, Yi Xiao

**Affiliations:** 1 School of Biological Science and Medical Engineering, Beihang University, Beijing, 100191 China; 2 National Key Laboratory of Human Factors Engineering, China Astronaut Research and Training Center, Beijing, 100094 China; 3 Beijing Advanced Innovation Centre for Biomedical Engineering, Beihang University, Beijing, 100191 China; 4 School of Logistics, Beijing Wuzi University, Beijing, 101149 China

**Keywords:** Neurophysiology, Circadian rhythms and sleep, Cognitive neuroscience

## Abstract

Cognitive abilities are impaired by sleep deprivation and can be recovered when sufficient sleep is obtained. Changes in alpha-band oscillations are considered to be closely related to sleep deprivation. In this study, power spectrum, source localization and functional connectivity analyses were used to investigate the changes in resting-state alpha-band activity after normal sleep, sleep deprivation and recovery sleep. The results showed that the global alpha power spectrum decreased and source activation was notably reduced in the precuneus, posterior cingulate cortex, cingulate gyrus, and paracentral lobule after sleep deprivation. Functional connectivity analysis after sleep deprivation showed a weakened functional connectivity pattern in a widespread network with the precuneus and posterior cingulate cortex as the key nodes. Furthermore, the changes caused by sleep deprivation were reversed to a certain extent but not significantly after one night of sleep recovery, which may be due to inadequate time for recovery sleep. In conclusion, large-scale resting-state alpha-band activation and functional connectivity were weakened after sleep deprivation, and the inhibition of default mode network function with the precuneus and posterior cingulate cortex as the pivotal nodes may be an important cause of cognitive impairment. These findings provide new insight into the physiological response to sleep deprivation and determine how sleep deprivation disrupts brain alpha-band oscillations.

## Introduction

Sleep is an indispensable physiological need in human life. Lack of sleep can lead to a decline in performance, and sleepiness is now widely believed to be one of the major causes of accidents^1,2^. Studies have recognized that sleep deprivation disturbs almost all specific processes of human behavior^3,4^. Sleep deprivation also negatively affects attention, memory, emotion and other advanced cognitive processes^5–7^. Of course, studies have also found that cognitive impairment caused by sleep deprivation can be recovered by adequate sleep^8,9^.

Growing findings indicate that changes in alpha-band oscillatory power are related to increased sleepiness. Researchers found that alpha power gradually decreased during the sleep onset transition^10^, and it was supposed that the decrease in alpha power during wakefulness may indicate an increase in sleep motivation^11^. In fact, a study has reported that subjective sleepiness during sleep deprivation shows an inverse correlation with alpha power^11^. In addition, trains of alpha waves become increasingly discontinuous during a prolonged transition from wakefulness to drowsiness, which is called "alpha power dropout"^12^. Studies have also found that decreased performance is accompanied by reduced alpha power after sleep deprivation, although the relationship was mostly studied in activating task situations, such as a vigilance task^13^. A coexistence relationship between decreased alpha activity and reduced performance (e.g., memory) has been well documented in pathology and aging studies^14^, which indicates that there is a substantial correlation between lower alpha activity and cognitive deficits. The decrease in alpha power appears to be associated with reduced activation of the limbic system in subcortical structures such as the brainstem, midbrain and hypothalamus because a positive correlation between local blood flow and resting-state alpha-band power has been found in these regions^11,15^. However, the cerebral cortices involved in alpha-band power changes after sleep deprivation have not been clearly specified or closely examined.

In addition to changes in brain oscillation power, many fMRI studies have shown that there is a disturbed coordination in distributed brain networks after sleep deprivation^16,17^. For example, studies have found that sleep deprivation not only reduces functional connectivity within the default mode network (DMN) but also reduces the anti-correlation between the DMN and its anti-correlated network^18–20^, indicating that sleep deprivation impairs coupling both within highly integrated cortical regions and between highly isolated networks^20^. It has been reported that changes in default mode activity after sleep deprivation may cause attention instability^21^. A similar case identified that the dissociation of functional connectivity within the DMN after sleep deprivation can impair sustained attention, thus affecting stable task performance^22^.

In sleep deprivation studies, many researchers used fMRI to examine changes in network coupling, but few used EEG recordings to measure changes in connectivity between brain regions. With the proposition that information communication in neural networks is mediated by synchronous neural activity^23–25^, the oscillatory mechanism of the connectivity changes revealed by EEG has attracted increasing interest. Alpha-band oscillation plays an important role in this framework because it is believed to reflect local and large-scale neuronal synchronization associated with several cognitive processes, such as top-down modulation, attention, inhibition and consciousness^26,27^. As mentioned above, alpha-band oscillation is closely related to sleep deprivation. We therefore hypothesized that cognitive impairment after sleep deprivation might be partially explained by electrophysiological changes in alpha-band oscillatory brain activity.

Sleep deprivation can damage a variety of cognitive functions, especially those functions associated with the frontal lobe^28,29^. In contrast, recovery sleep can change brain activity, thus improving performance on various cognitive tasks. Usually, after a night of recovery sleep, the changes in EEG and cognitive function return to the baseline level^13,30^. Other studies have found that one night may not be enough to fully recover the prefrontal lobe damage caused by sleep deprivation^31^. These studies suggest that recovery sleep has an organizing effect on cortical activity when subsequently awake.

The effect of sleep deprivation and recovery sleep on brain activation and functional connectivity in the resting-state alpha band remains unclear. Therefore, the purpose of this study was to investigate how sleep deprivation and recovery sleep could change alpha-band neural oscillations. We analyzed the power spectrum, subcortical source activation and functional connectivity in the resting-state alpha band to examine the differences after three sessions, e.g., normal sleep (NS), sleep deprivation (SD) and recovery sleep (RS). We hypothesized that (1) alpha-band power would decrease at both the scalp electrode level and cortical source level after sleep deprivation; (2) the connectivity of resting-state networks, especially that of the DMN, would be impaired by sleep deprivation; and (3) recovery sleep would reverse the damage caused by sleep deprivation at a certain level.

## Results

### Power spectrum comparisons

**Figure 1:**
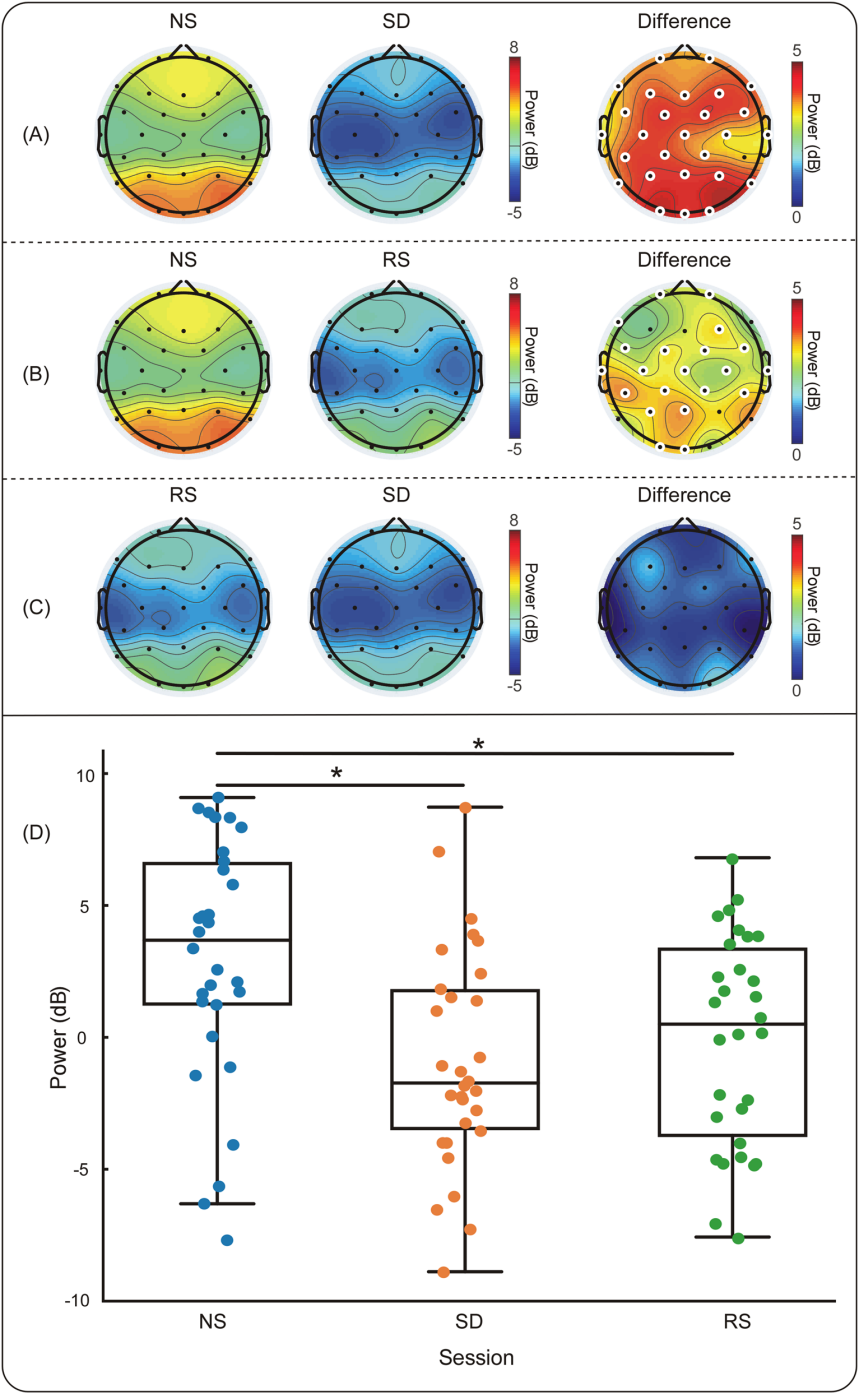
Alpha power differences. (**A**) Topographical distribution of NS and SD sessions. (**B**) Topographical distribution of NS and RS sessions. (**C**) Topographical distribution of RS and SD sessions. (**D**) Variability of the average power spectrum of the whole brain across subjects in the three sessions (NS vs. SD vs. RS, mean ± standard deviation: 3.133 ± 4.718 dB vs. − 0.771 ± 4.223 dB vs. 0.068 ± 4.061 dB). * p < 0.05. Enlarged white circles represent electrodes with significant differences. Abbreviations: NS, normal sleep; SD, sleep deprivation; RS, recovery sleep.

Power spectrum analysis was performed by analysis of variance (ANOVA) in a randomized block design and one-way repeated measures ANOVA. There was a significant difference across sessions (F(2, 126.71) = 6.468, *p* = 0.003); compared with NS sessions, SD sessions and RS sessions revealed a decrease in alpha power in most electrodes (both *p* < 0.017). The alpha power of each electrode in RS sessions was greater than that in SD sessions, but the differences were not significant (*p* > 0.017). Moreover, there were also significant differences in the mean power spectrum of the whole brain among different sessions (NS vs. SD vs. RS, mean ± standard deviation: 3.133 ± 4.718 dB vs. − 0.771 ± 4.223 dB vs. 0.068 ± 4.061 dB, F(2, 126.71) = 6.468, *p* = 0.003). The mean alpha power of NS sessions was significantly larger than that of SD (t = 3.286, *p* = 0.008) sessions and RS sessions (t = 2.654, *p* = 0.038), and there was no significant difference between SD and RS sessions (t = 0.775, *p* = 1.000). The power spectrum analysis results are presented in Fig. 1.

### Source location comparisons

**Figure 2:**
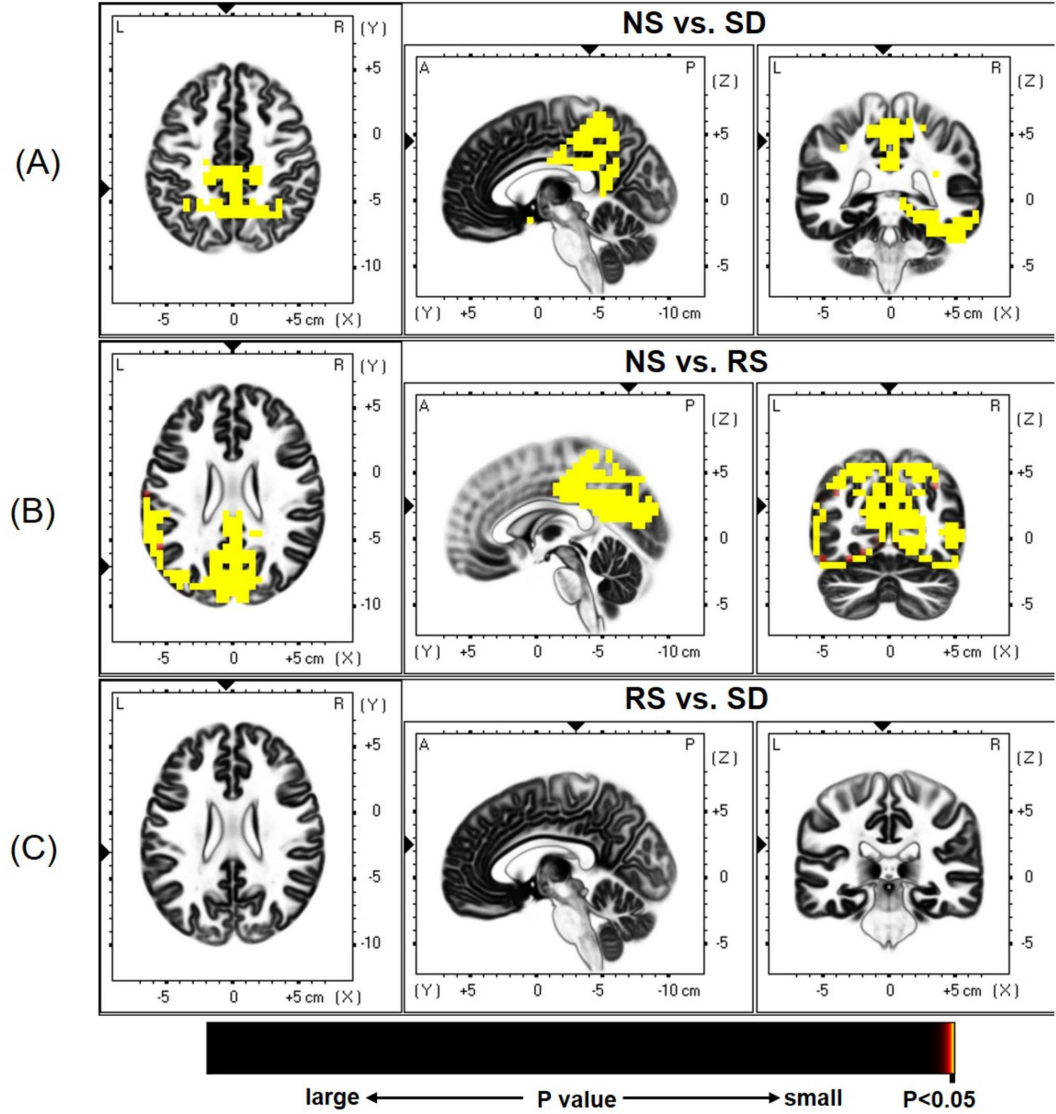
Differences in source activation between pairs of sessions. (**A**) NS session versus SD session; (**B**) NS session versus RS session; (**C**) RS session versus SD session. The significance level of activation contrast was set at *p* < 0.05.

Source location was analyzed by the statistical nonparametric mapping (SnPM) method^32,33^. As illustrated in Fig. 2, compared to NS sessions, SD sessions showed a widespread decrease in cortical activity, mainly including the cingulate gyrus, precuneus, paracentral lobule, and posterior cingulate cortex (BAs 31/7/5/23/30; t = 3.639; *p* < 0.01).

Similarly, compared to NS sessions, RS sessions revealed significant deactivation in the precuneus, cuneus, cingulate gyrus, paracentral lobule, and inferior parietal lobule (BAs 31/7/5/23/40; t = 3.635; *p* < 0.01).

However, no significant differences in activation were identified between NS and RS sessions (t = 2.829; *p* > 0.52).

### Functional connectivity comparisons

**Figure 3:**
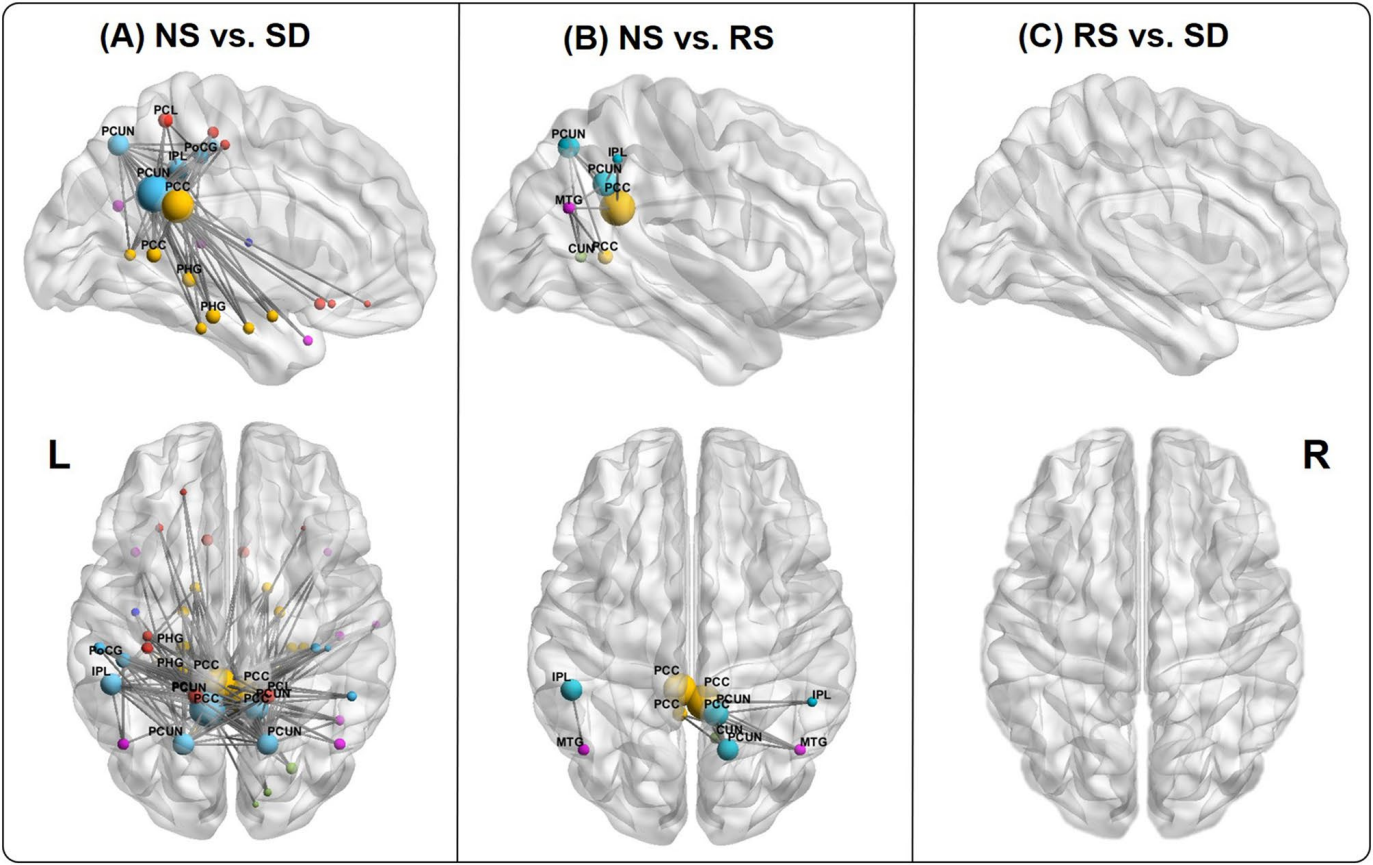
Alpha-band functional connectivity differences. (**A**) NS session versus SD session. Nodes with at least 14 connectivities are labeled, which contribute approximately 63% of the interactions to the network. (**B**) NS session versus RS session. All nodes are labeled because of the small number of connectivities. (**C**) RS session versus SD session. No significant connectivities were observed. Node size reflects the number of network connections. Abbreviations: PCC, posterior cingulate cortex; PCUN, precuneus; PoCG, postcentral gyrus; IPL, inferior parietal lobule; PCL, paracentral lobule; PHG, parahippocampal gyrus; MTG, middle temporal gyrus; CUN, cuneus. Color coding: parietal lobe, light blue; frontal lobe, red; limbic lobe, yellow; temporal lobe, purple; occipital lobe, green; insula, dark blue. The figure was visualized with the BrainNet Viewer (available at http://www.nitrc.org/projects/bnv/).

Functional connectivity was analyzed by the SnPM method**.** The functional connectivity of SD sessions, compared with that of NS sessions, exhibited significantly decreased alpha lagged linear connectivity in most cortical regions, especially in the parietal and limbic lobes (NS vs. SD, average connectivity values represent the mean ± standard deviation: 18.944 ± 2.447 vs 16.761 ± 2.782, t_max_ = 5.537, *p* < 0.01). The network mainly involved the precuneus, posterior cingulate cortex, paracentral lobule, inferior parietal lobule and parahippocampal gyrus (BAs 31/7/23/40/5/27/29); of these areas, the two nodes with the largest contribution were located in the precuneus and posterior cingulate cortex (Fig. 3A).

The functional connectivity of RS sessions was significantly decreased in the posterior cingulate cortex and middle temporal gyrus (BAs 23/39) compared with that of NS sessions (NS vs. RS: 18.944 ± 2.447 vs 17.763 ± 2.331, t_max_ = 4.446; *p* = 0.04) (Fig. 3B).

In addition, SD and RS sessions did not differ significantly in functional connectivity (SD vs. RS: 16.761 ± 2.782 vs 17.763 ± 2.331, t_max_ = 4.566, *p* = 0.07) (Fig. 3C).

## Discussion

In the present study, we utilized resting-state alpha-band EEG data to examine the effects of sleep deprivation and recovery sleep by comparing the differences among NS, SD and RS sessions. The alpha-band activation of SD sessions decreased over a wide range of cortical regions compared with that of NS sessions, especially in the precuneus, posterior cingulate cortex, cingulate gyrus, and paracentral lobule. Compared with NS sessions, the alpha-band functional connectivity of SD sessions decreased, with the precuneus and posterior cingulate cortex as the most critical nodes. In addition, there was a trend toward increased alpha-band activation and functional connectivity in RS sessions compared with SD sessions.

This study showed decreased alpha-band power in SD sessions compared with NS sessions, which was consistent with previous research^12,34,35^. Evidence has shown that there is a negative correlation between alpha power and subjective sleepiness^11^. The association between alpha power and sleepiness seems to be global, indicating that the attention and working memory involved in alpha-band oscillations may be related globally to sleepiness^11,14^.

In this study, the brain regions involved in decreased activation included the cingulate gyrus, precuneus, paracentral lobule, and posterior cingulate cortex (BAs 31/7/5/23/30), which are among the most often reported active regions after sleep deprivation in many fMRI studies^36,37^. Therefore, these cortices may play an important role in maintaining wakefulness. It is known that vitality after sleep deprivation is more negatively affected than after normal sleep, and the paraventricular lobule is considered to be negatively correlated with vitality activities^38^. This is in agreement with our current findings that SD sessions showed lower activation in the paracentral lobule compared with NS sessions. In accordance with the present results, previous studies have shown that the activity of the cingulate gyrus decreases with the extension of sleep deprivation, which is thought to reflect a decline in attention and executive function^39^. It is particularly notable that the precuneus and posterior cingulate cortex play a pivotal role in regulating the internal activities of the DMN^40,41^. Perturbations of DMN activity during wakefulness have been identified in many diseases accompanied by abnormal sleep, such as schizophrenia^42^ and anxiety disorders^43^, which may demonstrate that sleep modulates the DMN and maintains its function.

Furthermore, SD sessions showed reduced widespread functional connectivity compared with that of NS sessions. This result is in line with those of fMRI findings of sleep deprivation^18,44–46^. In addition, the results were also supported by previous studies that investigated functional connectivity in diseases with sleep abnormalities. Fingelkurts et al. reported that compared to control subjects, depression patients showed a desynchronization of the alpha band, mainly in the right anterior and left posterior brain areas^47^.

Moreover, it is noteworthy that the functional connectivity network changed after sleep deprivation and was mainly distributed in the limbic and parietal cortex. These regions have been found to be related to cognitive functions such as semantic processing^48^ and attention^49^ as well as working memory^50^. The reduced functional connectivity of these areas in the current results may indicate that these cognitive abilities are affected by sleep deprivation. The present analysis revealed that the precuneus and posterior cingulate cortex make the greatest contributions to the network, which are considered to be pivotal areas of the DMN and play an important role in mediating intrinsic activities^40^. Considering structural and functional connectivity^51,52^, our results suggested that the precuneus and posterior cingulate cortex are neural hubs damaged by sleep deprivation.

After a night of recovery sleep following sleep deprivation, alpha-band activation and functional connectivity did not return to normal levels, indicating that one night of sleep recovery cannot eliminate the damage caused by 36 h of sleep deprivation. In general, sleep has a recovery and organizing effect on the cortical activity of wakefulness^30,53^. Although the sleep recovery effect was not significant in our results, the difference between RS and SD sessions was smaller than the difference between NS and SD sessions, thus confirming the homeostatic regulation of sleep to a certain extent^54^.

The current alpha-band power spectrum results are consistent with the source localization results, which show that alpha-band power is decreased at both the scalp level and the source level after sleep deprivation. Similarly, the results of source location and functional connectivity are consistent, indicating that sleep deprivation greatly influences the DMN to which the precuneus and posterior cingulate cortex belong. Altogether, our complementary results showed that after sleep deprivation, the simultaneous decrease in cortical activation and connectivity weakened local processing and brain region cooperative processing. Based on the high coincidence of the alpha-band activation source and alpha-band functional connections of key node positions and the positive coupling of activation power and functional connectivity after sleep deprivation and recovery sleep, we cannot exclude the possibility that the alpha-band connectivities between the DMN and other brain regions may be modulated by oscillation power. This seems to be consistent with the idea that nerve synchronization influences functional integration^26,55^, indicating that power fluctuations in DMN alpha-band oscillations lead to cortical interaction changes.

Notably, there are several limitations to this study. First, no control group was set up to eliminate the possible influence of circadian rhythm changes on EEG recordings. The three EEG acquisition sessions in this study did not occur at the same time of day, and EEG data may be potentially affected by participants’ circadian rhythms. Fortunately, studies have confirmed that EEG changes caused by sleep deprivation are hardly affected by circadian rhythms^56,57^. Second, the spatial resolution of the source localization and connectivity analysis was not very high. The spatial resolution of EEG sources increases with the number of electrodes, so high electrode density recording is more reliable in EEG rhythm source analysis. The use of a standard MRI template instead of individual MRIs for source localization further decreases the possible spatial resolution. Third, all of our subjects were men, so the results should be extrapolated to women with some caution. Fourth, the current study examined only EEG changes in the alpha band caused by sleep deprivation and recovery sleep, while possible changes in other frequency bands were not taken into account.

This study found that resting-state alpha-band activation and functional connectivity decreased after sleep deprivation, and these changes were not significantly reversed after one night of sleep. Our results reflect the electrophysiological evidence of resting-state alpha-band deactivation and dysconnectivity in extensive cerebral cortices, especially in the DMN with the precuneus and posterior cingulate cortex as pivotal regions. Changes in these regions may be associated with cognitive impairment caused by sleep deprivation.

## Methods

### Participants

The sample size was calculated using G*Power^58^. A total sample size of 29 participants was required to obtain a standard effect size of 0.25 (which is considered medium according to Cohen^59^) and to achieve a power of 0.8, with α error probability of 0.05. To avoid a reduction of statistical power due to potential dropouts, 30 graduate students (age range: 22–26 years; mean = 23.8; standard deviation = 1.4) were recruited from Beihang University. All subjects were male, right-handed, had no sleep disorder, and had no self-reported history of mental illness or medication history of the central nervous system. All participants provided written informed consent, and the study was approved by the Research Ethics Board of Beihang University. All methods were performed in accordance with the relevant guidelines and regulations.

### Protocol

**Figure 4:**
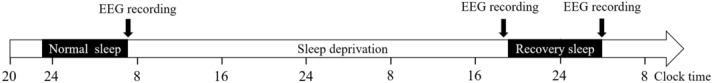
Study protocol.

The experimental protocol consisted of a normal night, a sleep deprivation period, and a recovery night. Participants entered the laboratory the day before the experiment and did not leave until all sessions were completed. On the normal night, subjects obtained approximately 8 h of normal sleep, followed by 36 h of sleep deprivation. On the following recovery night, subjects underwent recovery sleep, which was not limited to 8 h but could not be extended past 10 h (Fig. 4). The subjects refrained from caffeine, alcohol, and strenuous exercise a day before and during the entire experiment. During the SD period, participants were supervised by study staff to ensure they were awake. Resting-state EEG after the three sessions (NS, SD and RS) was recorded. During each EEG recording, participants were instructed to close their eyes but stay awake and think of nothing in particular.

### EEG recording and processing

The experiment was carried out in a dimly lit, sound-attenuated chamber. Resting-state EEG data were recorded for 3 min from participants while they were awake, were comfortably seated and had their eyes closed. EEG data were acquired from 32 electrodes placed according to the international 10–20 system using an elastic cap (actiCAP, Brain Products GmbH, Gilching, Germany). EEG recordings were accomplished by using Brain Vision Recorder software (Brain Products, Germany). The sampling rate was set at 1000 Hz, and the impedance of the EEG signal was kept below 5 kΩ. Vertical and horizontal electro-oculograms were recorded with electrodes placed below and on the outer canthus of the left eye and used to correct the EEG recordings for eye movement artifacts.

EEG preprocessing was performed with MATLAB R2017 (MathWorks, Natick, MA). The raw data were resampled to 250 Hz and rereferenced to the average reference. EEG data were treated with an 8–12 Hz bandpass filter. Then, EEG data were divided into 2-s epochs. Off-line artifact rejection was performed by visual inspection to eliminate the effects of eye/muscle movements. Independent component analysis was further conducted to eliminate ocular and prominent muscle artifacts^60^.

### Power spectral analysis

Absolute power was calculated using Welch’s periodogram method in MATLAB, with nonoverlapping Hamming windows of 2 s^61,62^. The log-transformed power spectra of the alpha band (8–12 Hz) were calculated, which was followed an average power computation.

### EEG source localization analysis

Underlying cortical sources of the alpha band were estimated using the sLORETA software package^63–65^ (available at http://www.uzh.ch/keyinst/loreta). Source localization was performed in the frequency domain to compute the cortical three-dimensional distribution of neuronal activity. Cross-spectral matrices for each subject were computed and then averaged as the input for the source analysis. The solution space corresponded to 6239 voxels at a 5 mm spatial resolution. Source activations were estimated using a head model based on the Montreal Neurological Institute (MNI) 152 standard template^66^.

### Functional connectivity analysis

Functional connectivity was computed by eLORETA software (available at http://www.uzh.ch/keyinst/loreta) on 84 regions of interest (ROIs) defined according to the 42 Brodmann areas (BAs) in the left and right hemispheres. The ROIs were determined by 30 electrodes (Fp1, Fp2, F3, F4, F7, F8, FC1, FC2, FC5, FC6, Fz, C3, C4, Cz, CP1, CP2, CP5, CP6, T7, T8, TP9, TP10, P3, P4, P7, P8, Pz, O1, O2, Oz). The signal at each ROI was the average electrical neuron activity of all voxels in the ROI^67^. Among the eLORETA current density time series of the 84 ROIs, lagged linear connectivity^68–70^ was computed between all possible pairs of the 84 ROIs for the alpha band for each subject. Physiological measures of lagged linear connectivity were used, instead of classical connectivity-type measures that mostly indicate common sources and not true connectivity. Such connectivity addresses instantaneous, nonphysiological signal contamination due to volume conduction^68,71^ by calculating the sum of lagged dependence and instantaneous dependence.

### Statistical analysis

In the comparison of the power spectrum, source localization and functional connectivity among the sessions, pairwise comparisons were performed to test the difference between each two sessions, i.e., NS versus SD, RS versus SD, and NS versus RS.

For the power spectrum analysis, the log-transformed absolute power has been shown to be approximately normal distribution^72^. Differences between sessions were assessed by ANOVA in a randomized block design with each electrode considered a random block. Post hoc analysis was performed using paired t-test with a Bonferroni correction for multiple comparisons (α = 0.05/3 = 0.017), and an FDR correction was further applied for pairwise electrode comparisons^73,74^. In addition, the average power of all electrodes was also calculated using one-way repeated measures ANOVA to compare the differences among sessions, with Geisser-Greenhouse adjustments for nonsphericity and Bonferroni post hoc tests, where appropriate.

For the source localization analysis, based on the log-transformed current source density power determined by sLORETA, we evaluated the difference in cortical source activation between sessions by an independent F ratio test of each voxel. Statistical analysis was performed using the SnPM method implemented in sLORETA software. The method utilized Fisher’s random permutation test with 5000 randomizations to correct for multiple comparisons.

For the functional connectivity analysis, tests were conducted using eLORETA to examine all connectivities between 84 ROIs (3486 connectivities) in the alpha band. In addition, we also applied the SnPM method based on the "maximum statistic" to correct for multiple comparisons.
